# Identification and Manipulation of Atomic Defects in Monolayer SnSe

**DOI:** 10.1021/acsnano.4c04789

**Published:** 2024-09-05

**Authors:** Chengguang Yue, Zhenqiao Huang, Wen-Lin Wang, Zi’Ang Gao, Haicheng Lin, Junwei Liu, Kai Chang

**Affiliations:** † Beijing Academy of Quantum Information Sciences, Beijing 100193, China; ‡ Department of Physics, Hong Kong University of Science and Technology, Clear Water Bay, Hong Kong

**Keywords:** atomic defects, monolayer SnSe, two-dimensional ferroelectricity, local density of states, molecular beam epitaxy, scanning tunneling microscopy

## Abstract

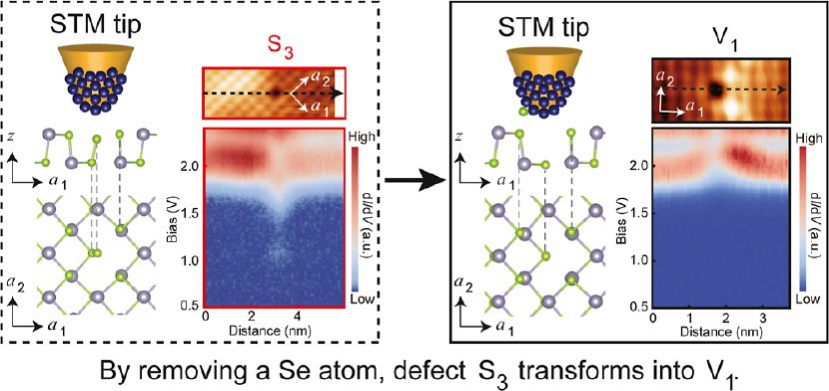

SnSe, an environmental-friendly group-IV monochalcogenide semiconductor, demonstrates outstanding performance in various applications ranging from thermoelectric devices to solar energy harvesting. Its ultrathin films show promise in the fabrication of ferroelectric nonvolatile devices. However, the microscopic identification and manipulation of point defects in ultrathin SnSe single crystalline films, which significantly impact their electronic structure, have been inadequately studied. This study presents a comprehensive investigation of point defects in monolayer SnSe films grown via molecular beam epitaxy. By combining scanning tunneling microscopy (STM) characterization with first-principles calculations, we identified four types of atomic/molecular vacancies, four types of atomic substitutions, and three types of extrinsic defects. Notably, we have demonstrated the ability to convert a substitutional defect into a vacancy and to reposition an adsorbate by manipulating a single atom or molecule using an STM tip. We have also analyzed the local atomic displacement induced by the vacancies. This work provides a solid foundation for engineering the electronic structure of future SnSe-based nanodevices.

Group-IV monochalcogenides are a family of semiconductors with orthorhombic lattices that resemble staggered black phosphorus. Their relatively low crystalline symmetry makes their physical properties highly tunable. Among them, SnSe, with a moderate bandgap,^1−3^ is utilized in various applications including photodetectors,^4^ solar cells,^5^ photocatalysis,^6^ supercapacitors,^7^ gas sensors,^8^ memristors,^9^ thermoelectric materials,^10,11^ and anode materials for batteries.^12^ Recent studies have identified topological crystalline insulating phases in rocksalt Pb_1–*x*_Sn_*x*_Se single crystals and SnSe thin films.^13−16^ SnSe is also renowned for its thermoelectricity, attributed to robust anharmonicity and outstanding in-plane electrical transport,^10,11^ especially in the β-SnSe phase. Notably, α-SnSe demonstrates two-dimensional (2D) ferroelectricity,^17−20^ which allows for the switchable in-plane spontaneous polarization at the monolayer level and at room temperature.^17^ Since the properties and applications above are highly sensitive to the electronic structure and chemical potential of SnSe, understanding the impact of point defects holds significant interest.

Extensive research on SnSe’s point defects is primarily driven by their significant impact on the material’s exceptional thermoelectric properties. Density functional theory (DFT) modeling has extensively addressed intrinsic defects such as vacancies (V_Sn_, V_Se_), antisites (Sn_Se_, Se_Sn_), and interstitials (Sn_i_, Se_i_), as well as extrinsic defects, in numerous studies.^21−27^ Notably, charge defects such as V_Sn_ and V_Se_, which increase carrier concentration, are key to achieving a high power factor.^21^ These calculations identify V_Sn_ as the main factor in SnSe’s intrinsic p-type conductivity, attributed to its relatively low formation energy and shallow defect energy level.^22−25^ Experimental observations utilizing scanning tunneling microscopy (STM) have revealed the vacancies of Sn and Se atoms, as well as bunched vacancies involving multiple atoms (referred to as “multivacancies”), through atom-resolved images and differential conductance (d*I*/d*V*) spectra, from which the origin of p-type doping from Sn vacancies was affirmed.^25,28,29^ Furthermore, the structures of V_Sn_, Se_i_, and multivacancies have been resolved using scanning transmission electron microscopy.^29−32^ Studies on the formation of V_Sn_ and V_Se_ at different annealing temperatures were conducted using positron annihilation spectroscopy combined with transport measurements.^33^ Nevertheless, there remains a lack of systematic research focusing on the local density of states (LDOSs) of all types of intrinsic defects in SnSe. Moreover, previous studies on SnSe defects were predominantly static and lacked exploration into the transition between different types of defects, particularly in regards to controlled conversion.

Here, by combining low-temperature STM with DFT calculations, we have conducted a comprehensive analysis of the atomic and electronic structures of point defects in a single van der Waals monolayer (two atomic layers) of SnSe. This includes 8 types of intrinsic defects and 3 types of extrinsic defects. Furthermore, we demonstrated the capability to convert a substitution defect into a vacancy through the manipulation of the electric field between the STM tip and the sample surface. This study provides essential insights for future explorations in SnSe-based devices.

## Results and Discussion

**Figure 1:**
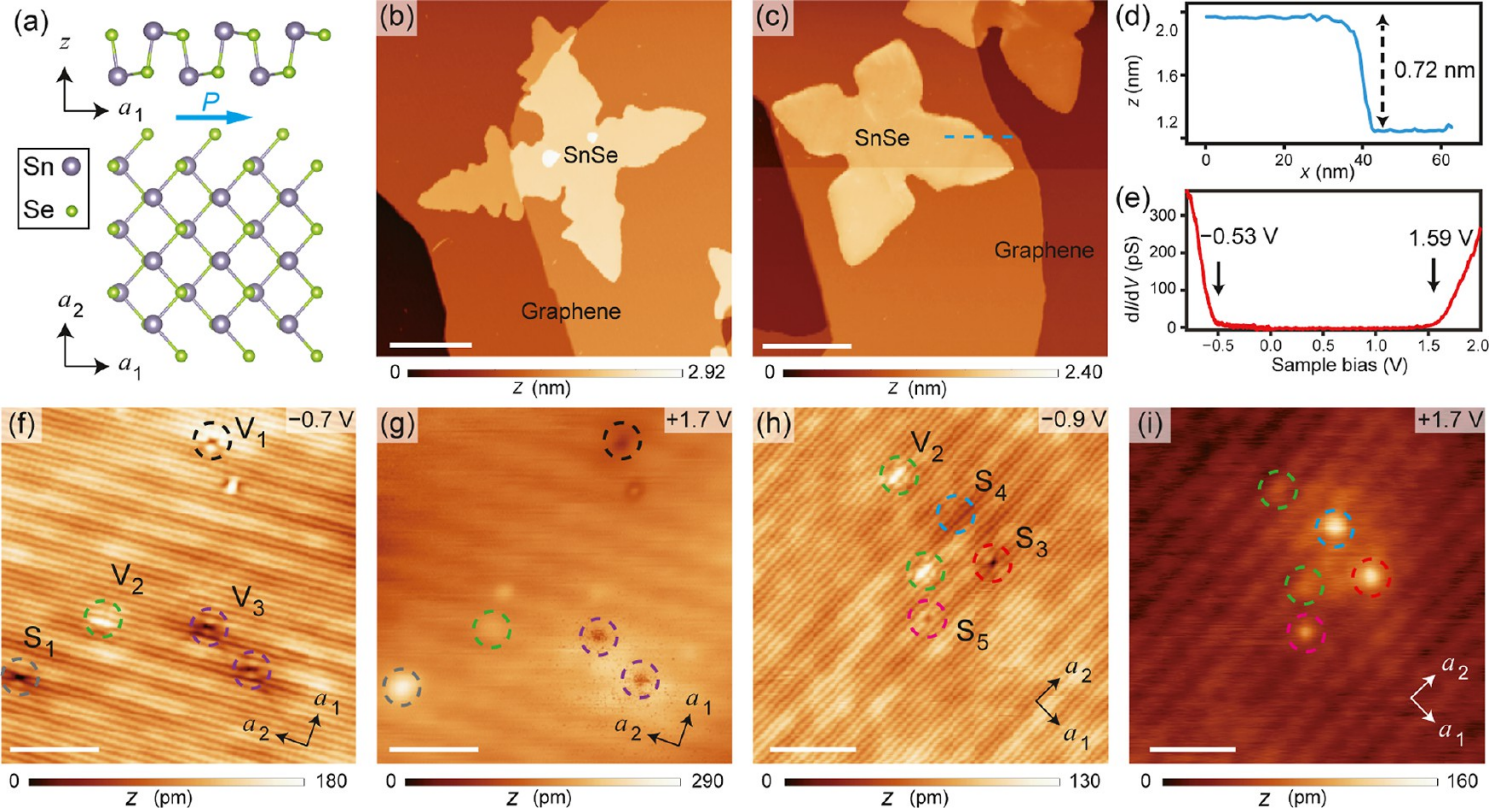
Point defects in monolayer SnSe. (a) Side view (upper panel) and top view (lower panel) of the lattice structure of monolayer SnSe. (b,c) Typical STM topography images of monolayer SnSe grown with the substrate kept at room temperature (b) and 100 °C (c), and subsequently annealed at 100 °C for 30 min. (d) The apparent height profile was extracted along the dashed line in (c). (e) d*I*/d*V* spectrum obtained from a defect-free area on the surface of monolayer SnSe. The arrows indicate the energies of the CBM and VBM. (f–i) Typical point defects in monolayer SnSe. Dashed circles in different colors are used to mark the type of defects. (f)/(g) and (h)/(i) are pairs of images acquired at the same positions but with different *V*_s_. Tunneling parameters: (b) *V*_s_ = – 1.0 V, *I*_t_ = 5 pA; (c) *V*_s_ = + 1.7 V, *I*_t_ = 10 pA; (e) *V*_s_ = + 2.0 V, *I*_t_ = 50 pA, the sinusoidal modulation voltage *V*_OSC_ = 20 mV for positive *V*_s_, and *V*_s_ = – 0.8 V, *I*_t_ = 50 pA, *V*_OSC_ = 8 mV for negative *V*_s_; (f) *V*_s_ = – 0.7 V, *I*_t_ = 5 pA; (g) *V*_s_ = + 1.7 V, *I*_t_ = 5 pA; (h) *V*_s_ = – 0.9 V, *I*_t_ = 100 pA; (i) *V*_s_ = + 1.7 V, *I*_t_ = 200 pA. The scale bars in (b,c) are 80 nm, while those in (f–i) are 5 nm.

As previously reported, the lattice structure of SnSe varies with temperature and epitaxial conditions, alternating between an orthorhombic α phase (space group *Pnma*) or β phase (space group *Cmcm*), or a rocksalt phase (space group Fm3̅m).^3,16^ In this study, all monolayer SnSe samples crystallize in the α phase, which has a spontaneous in-plane polarization along its ***a***_1_ direction. This polarization reduces the crystalline symmetry to *Pnm*2_1_, as illustrated in Figure 1a.

Our investigation commenced with synthesizing monolayer SnSe films on graphene by directly depositing SnSe molecules on a graphitized 4H–SiC(0001) substrate at either room temperature (Figure 1b) or 100 °C (Figure 1c). We deliberately keep the substrate temperature low to increase the concentration of point defects. The apparent height of the monolayer, consisting of two atomic layers, ranges between 0.72 and 0.78 nm, depending on the applied sample bias voltage *V*_s_. Annealing at 250 °C leads to the formation of square-shaped nanoplates with a lower defect concentration, as reported in a previous study.^17^ The d*I*/d*V* spectrum, acquired at defect-free areas, reveals a band gap of 2.12 eV, with the conduction band minimum (CBM) at 1.59 eV and the valence band maximum (VBM) at – 0.53 eV (Figure 1e), in agreement with previous measurements.^17^ The band gap of monolayer SnSe is significantly larger than those reported in bulk SnSe, which range between 0.86 and 1.35 eV.^3^ This difference is attributed to the quantum size effect in the atomically thin nanoplates, where the bulk electronic bands further quantize into 2D quantum well states with parabolic dispersion. As the material’s thickness decreases, the energy separation between the apexes of these parabolic bands increases, leading to a corresponding rise in the band gap. Our measurement displays a quick drop of band gap size from 2.12 eV to approximately 1.4 eV as the thickness increases from one monolayer to six monolayers.

Figure 1f–i exhibit atom-resolved STM topography images of the point defects in monolayer SnSe films. The crystalline orientation can be easily identified from the moiré stripes (Figure S1), with the ***a***_1_ and ***a***_2_ axis being perpendicular and parallel to the stripes, respectively.^17^Figure 1f,g show the same group of point defects, resolved at *V*_s_ below the VBM and above the CBM, corresponding to the filled and empty states, similar as in Figure 1h,i. Only the Sn sublattice is resolved at both positive and negative *V*_s_ because the Sn atoms are lifted compared to the Se atoms on the surface of this staggered black phosphorus lattice (Figures 1a and S2).^24^ The atoms are usually more clearly resolved at a negative *V*_s_, probably due to differences in local atomic orbitals near the CBM and VBM. The point defects within a single image act as references for each other, enabling precise positioning of each point defect, irrespective of the resolution of atomic lattices.

The comprehensive studies involving STM topography, d*I*/d*V* spectra, and DFT calculations enable us to identify the atomistic configurations of various point defects, especially those with similar structures. For instance, we can distinguish the same type of vacancy defects occurring in different atomic layers, as well as the same type of substitution defects with minor variations in atomic structure. Consequently, we have cataloged 8 types of intrinsic point defects and 3 types of extrinsic point defects, as listed in Table 1. These include 4 types of atomic/molecular vacancies (V_1_–V_4_), 4 types of Se antisite substitution of Sn (S_1_–S_4_), 1 type of Pb-substitution of Sn (S_5_), and 2 types of adsorbates (A_1_, A_2_). The justification for these defects is detailed below.

**Table 1:** Types of Point Defects and Their Notations

defect type	notation
Sn vacancy in SAL	V_1_
Sn vacancy in BAL	V_2_
combined defect a Sn vacancy in SAL and a Se vacancy in BAL	V_3_
combined defect a Se vacancy in SAL and a Sn vacancy in BAL	V_4_
Sn-substituted by Se in SAL (configuration 1)	S_1_
Sn-substituted by Se in BAL (configuration 1)	S_2_
Sn-substituted by Se in SAL (configuration 2)	S_3_
Sn-substituted by Se in BAL (configuration 2)	S_4_
Sn-substituted by Pb in SAL	S_5_

**Figure 2:**
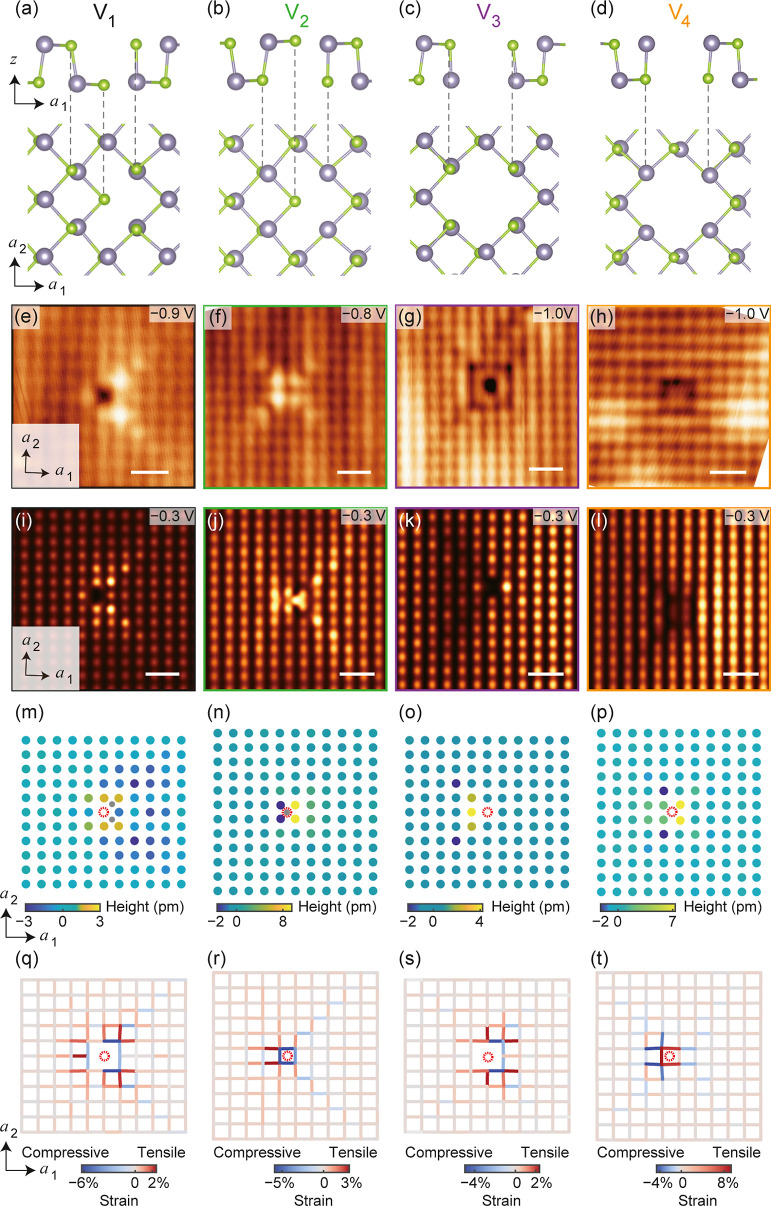
Atomic structures and the topographic appearances of vacancy defects V_1_–V_4_. (a–d) Side view (upper panels) and top view (lower panels) of the atomic structures of defects V_1_–V_4_, respectively. (e–h) Atom-resolved topography images of defects V_1_–V_4_. All scale bars correspond to 1 nm. The tunneling parameters for each image are specified as follows: (e) *V*_s_ = – 0.9 V, *I*_t_ = 10 pA; (f) *V*_s_ = – 0.9 V, *I*_t_ = 10 pA; (g) *V*_s_ = – 0.8 V, *I*_t_ = 30 pA; (h) *V*_s_ = – 1.0 V, *I*_t_ = 30 pA. (i–l) Simulated atom-resolved STM topography images of all vacancies. The sample bias voltages used in simulation are −0.3 V for all. The simulations of the topography images have set the VBM equal to the Fermi level. Distribution of height (m–p) and interatomic distances (q–t) of Sn sublattice within SAL around defects V_1_–V_4_. The red dashed circles indicate the positions of Sn and Sn–Se vacancies. The smaller dots are Se atoms with a height higher than Sn atoms in the (m) and (n).

For the vacancy defects, we attribute V_1_/V_2_ to atomic Sn vacancies in the surface/bottom atomic layer (SAL/BAL), and V_3_/V_4_ to vertical molecular Sn–Se vacancies at the Sn/Se site in SAL, respectively (Figure 2a–d). These assignments correspond with the features in STM topography images: V_1_ and V_3_ show a suppression in LDOSs at a Sn site in SAL during tunneling into both filled and empty states (Figures 2e,g; S5), while V_2_ and V_4_ are located at the center of the four nearest Sn atoms in the SAL. Noncentrosymmetric features, induced by the in-plane polarization along ***a***_1_, are observed in these defects, especially in V_1_ and V_2_, near the VBM. Bright branches indicating higher LDOSs extend from V_1_ along the [11̅0] and [110] directions, similar to features reported in early STM studies on surficial Sn vacancies in bulk SnSe.^25,28,29^ The four nearest Sn atoms on top of V_2_ are highlighted, with the two atoms in the direction antiparallel to in-plane polarization appearing brighter. In comparison, V_3_ and V_4_ introduce less significant changes to the LDOSs in SAL, likely due to their charge-neutral nature. According to previous studies, Sn vacancies are a primary source of intrinsic p doping in SnSe,^22,23,25,28,29,31^ while SnSe molecule vacancies do not introduce additional charge carriers. The STM topography features, including noncentrosymmetric appearances, are well reproduced by the DFT calculations (Figure 2i–l). Noticeably, the simulated topography of V_2_ shows enhanced LDOS at the site of a Se atom in SAL; this feature is also experimentally observed in the d*I*/d*V* mapping images when *V*_s_ is set around −1.0 V (Figure S3).

**Figure 3:**
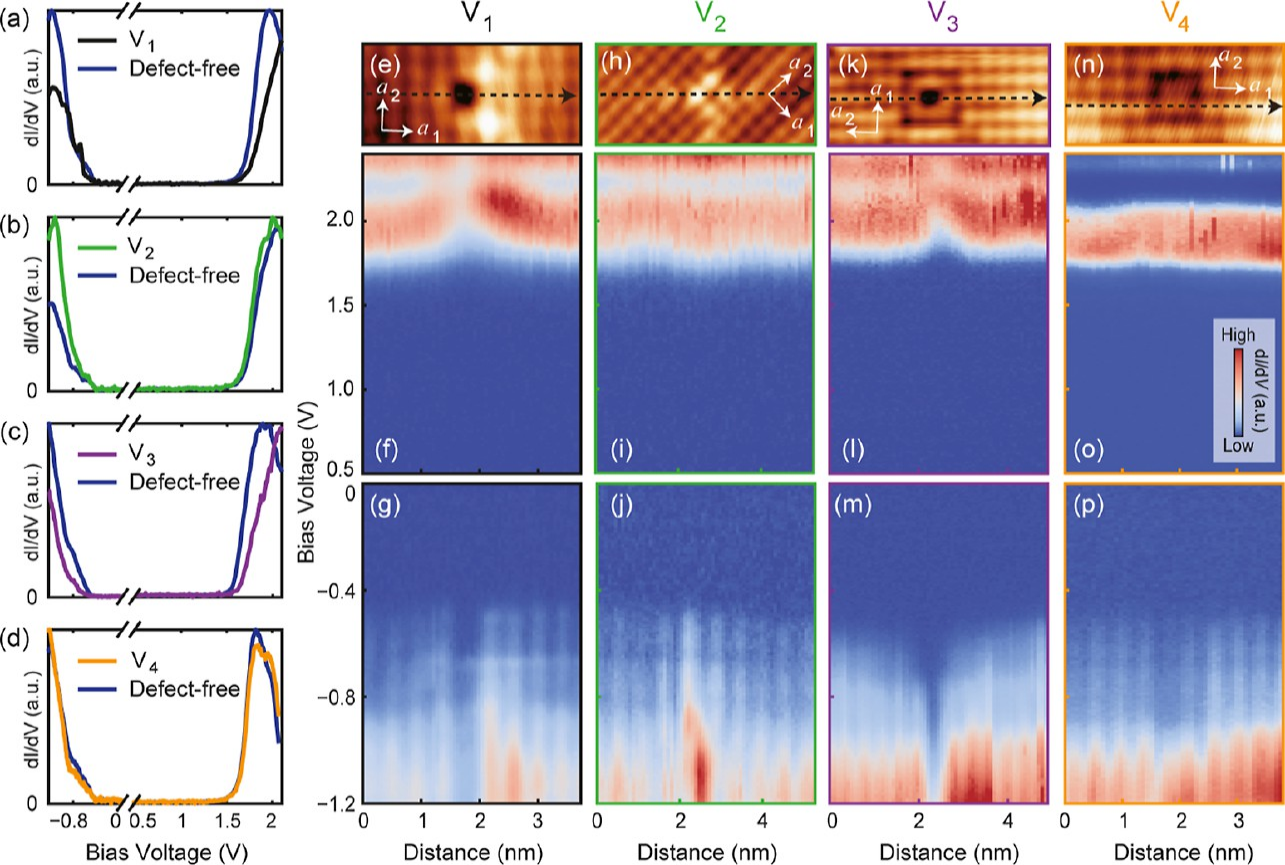
d*I*/d*V* spectra of defects V_1_–V_4_. (a–d) Comparison of the d*I*/d*V* spectra acquired right at the defect position and those from defect-free areas. (f,g,i,j,l,m,o,p) Spatially resolved d*I*/d*V* spectra of V_1_–V_4_, obtained along the dash arrows in (e,h,k,n), respectively. Because the d*I*/d*V* intensity of conduction and valence bands are significantly different, the spectra above and below the Fermi level were measured under different tunneling parameters for clarity: (f,l) *V*_s_ = + 2.4 V, *I*_t_ = 200 pA, *V*_OSC_ = 24 mV; (i,o) *V*_s_ = + 2.4 V, *I*_t_ = 100 pA, *V*_OSC_ = 24 mV; (g) *V*_s_ = – 1.5 V, *I*_t_ = 200 pA, *V*_OSC_ = 15 mV; (j,p) *V*_s_ = – 1.5 V, *I*_t_ = 100 pA, *V*_OSC_ = 15 mV; (m) *V*_s_ = – 1.2 V, *I*_t_ = 200 pA, *V*_OSC_ = 12 mV.

Based on the relaxed atomic structures obtained from DFT calculations, we have extracted the lattice distortion map at each atomic site (Figure 2m–t). For all the four types of vacancy defects, the induced noncentrosymmetric distortion extends over 2–3 Sn atom sites from the defect center, aligning with the experimental results. Interestingly, the enhanced LDOSs around V_1_ and V_2_ do not derive from an increase in the corresponding Sn atoms’ height in SAL. In contrast, many of these Sn atoms shift downward. Therefore, the primary cause of LDOS enhancement appears to be changes in electronic states, which is further characterized by the d*I*/d*V* spectra obtained at these defects (Figure 3). None of these defects introduce observable states inside the semiconducting gap of monolayer SnSe. Directly at the defect site, V_1_ and V_3_ show a suppression in LDOSs at both their CBM and VBM (Figure 3a,c,e–g,k–m), while the CBM and VBM of V_2_ and V_4_ are hardly affected (Figure 3b,d,h–j,n–p). This can be understood as both V_1_ and V_3_ involve the absence of a Sn atom in SAL, while only Sn atoms can be resolved in STM topography images. Notably, V_2_ shows significant LDOS enhancement when *V*_s_ is set below −0.8 V (Figure 3b,j), consistent with the DFT simulation (Figure 2j) and d*I*/d*V* mapping (Figure S3). Interestingly, as a molecular vacancy, V_4_ hardly affects the LDOS of the CBM (Figures 3o and S5), which is consistent with its charge neutrality, and its position sitting in between four Sn atoms.

The spatial oscillations in Figure 3g–p correspond to the atomic corrugation of the SnSe lattice. When acquiring d*I*/d*V* spectra along the ***a***_1_ direction of SnSe, the period of oscillation is about 4.4 Å; while along the [11] direction, it extends to about 6.1 Å. These measurement values are consistent with the lattice parameters of SnSe. Significantly, these spatial oscillations can only be observed under negative bias voltage. This phenomenon is also evident in the topography images, where atoms appear more distinctly at negative *V*_s_ due to the less localized electronic states around the CBM. It is also worth noting that, although the VBM mainly consists of the orbitals of Se atoms, the atoms resolved at negative bias voltage are still Sn. This is attributed to the Sn sublattice being slightly higher than the Se at the surface, as reported in previous studies.^17,24^

We noted that Sn atom vacancies are broadly reported in STM studies of bulk SnSe crystals,^25,28,29^ however, reports of Sn–Se molecular vacancies are rare. This is probably because our SnSe films were grown from the deposition of SnSe molecules, rather than synthesizing them from single Sn and Se elements. Different from the growth of bulk crystals, the molecular beam epitaxy (MBE) growth of thin films can be a process far away from thermal equilibrium, because the latter usually happens at a much lower temperature and in a much shorter time than the former. For instance, our growth of monolayer SnSe nanoplates happened at a substrate temperature of 300–370 K, much lower than SnSe’s melting point, and the growth only took several minutes. Therefore, growth kinetics largely affect the crystalline structure and the defect types in the MBE grown films.^34−37^ When directly depositing SnSe molecules, the formation of atomic defects involves breaking the bond between Sn and Se atoms in a molecule (our calculation yields a bonding energy of 5.04 eV), and forming new bonds with the existing film. Therefore, only the atomic defects with low enough formation energy can appear, such as Sn vacancies and the Se-substitution of Sn. The latter even has negative formation energy in a monolayer SnSe film (Table 2), implying that it would automatically appear as long as excess Se exists. However, the formation energy of Se vacancies in monolayer SnSe is 0.778 eV according to our calculations, much higher than that of Sn vacancies (0.382 eV). On the other hand, this growth kinetics increases the possibility of forming SnSe molecular vacancies, because this process only involves the deposition of whole molecules, while does not need to break the bond inside a molecule. In fact, similar molecular vacancies have also been observed in other materials that were grown from the evaporation of a single compound, such as those in CdTe crystals.^38^

**Table 2:** Formation Energies and Densities of the Intrinsic Point Defects

		defect density (10^10^ cm^–2^)	
defect type	formation energy (eV)	deposit at RT	deposit at 100 °C
V_1_	0.382 ± 0.005	2.89	2.87
V_2_		19.86	10.75
V_3_	4.407 ± 0.002	2.89	3.58
V_4_		0.00	0.72
S_1_	–2.680 ± 0.028	17.33 (total number of S_1_∼S_4_)	19.35 (total number of S_1_∼S_4_)
S_2_			
S_3_	–2.358 ± 0.005		
S_4_			

**Figure 4:**
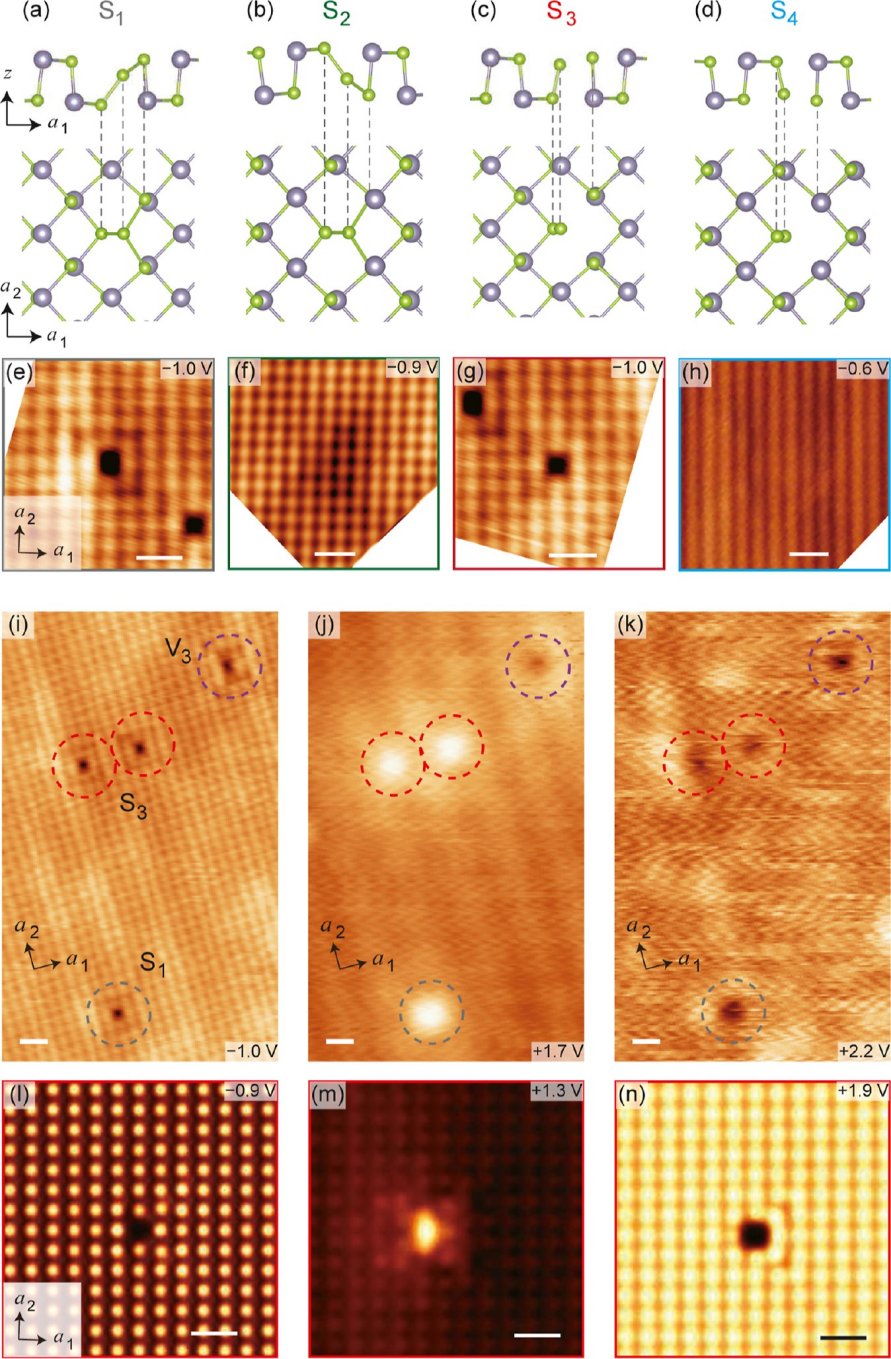
Atomic structures and the topographic appearances of substitution defects S_1_–S_4_. (a–d) Side view (upper panels) and top view (lower panels) of the atomic structures of defects S_1_–S_4_, respectively. (e–h) Atom-resolved topography images. The features of defects S_2_ and S_4_ are clearer at positive *V*_s_ (see Supporting Information). (i–k) STM images of defects S_1_ and S_3_ at different sample bias voltages. The defects S_1_, S_3_ and V_3_ are indicated by gray, red and purple dash circles respectively. (l–n) Corresponding simulated STM images of defect S_3_. The tunneling parameters for each image are specified as follows: (e) *V*_s_ = – 1.0 V, *I*_t_ = 30 pA; (f) *V*_s_ = – 0.9 V, *I*_t_ = 50 pA; (g) *V*_s_ = – 1.0 V, *I*_t_ = 30 pA; (h) *V*_s_ = – 0.6 V, *I*_t_ = 10 pA; (i) *V*_s_ = – 1.0 V, *I*_t_ = 30 pA; (j) *V*_s_ = + 1.7 V, *I*_t_ = 10 pA; (k) *V*_s_ = + 2.2 V, *I*_t_ = 10 pA. All scale bars correspond to 1 nm.

Besides the vacancy defects, our findings regarding the antisite substitution defects (S_1_–S_4_) are more intriguing. In these defects, two distinct atomistic configurations emerge when a Se atom substitutes a Sn atom, as illustrated in Figure 4a–d. At a positive *V*_s_ close to the CBM, all antisite substitution defects appear as bright spots in STM topography images. However, at higher *V*_s_, their apparent heights are suppressed, resulting in a dip (Figures 4j,k, S6). Conversely, at a negative *V*_s_ close to the VBM, the two types of defects in SAL, S_1_ and S_3_, show a dip at the original Sn sites that were substituted (Figure 4e,g). However, the other two types in BAL, S_2_ and S_4_, hardly show any features at negative *V*_s_ (Figure 4f,h). A comparison between experimental topography images and the DFT simulated images shows a strong correlation across different ranges of *V*_s_ (Figure 4i–n).

**Figure 5:**
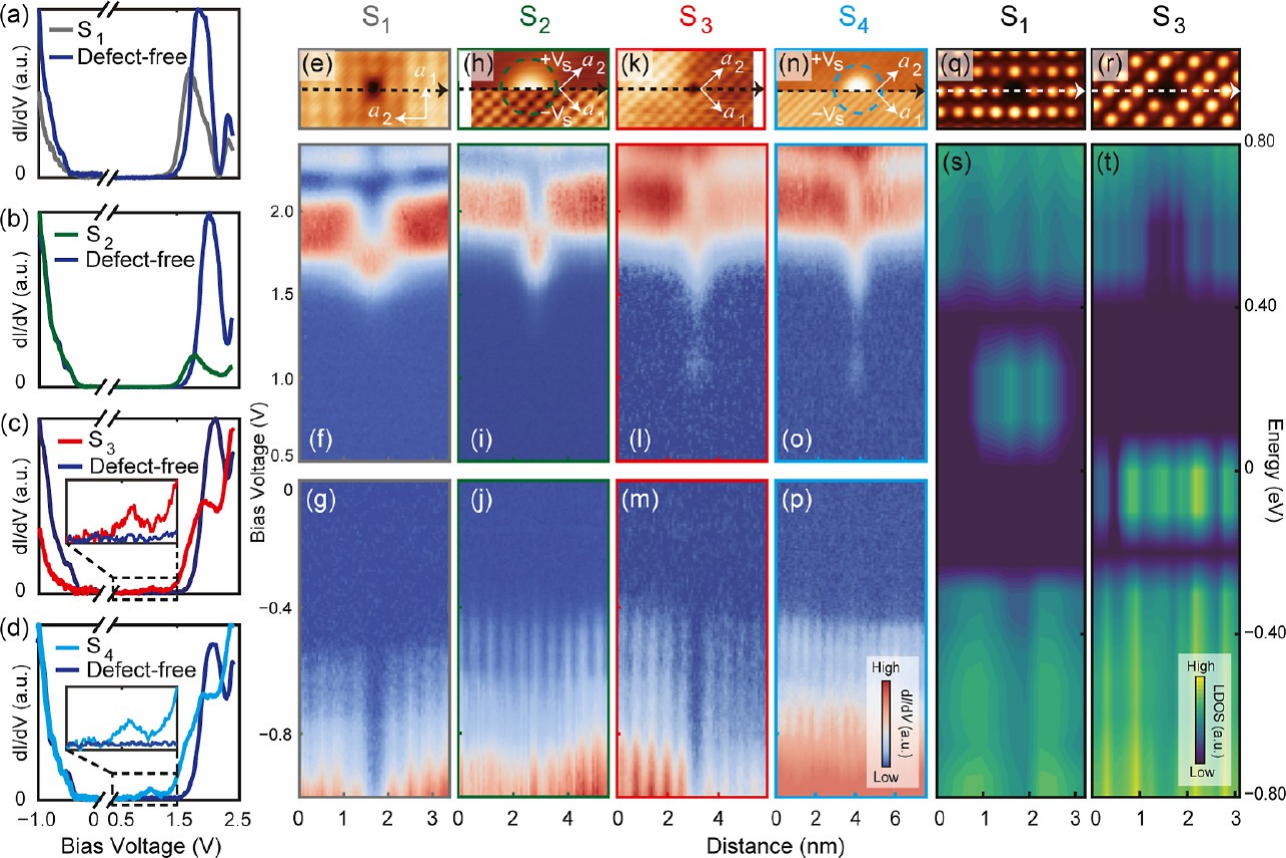
d*I*/d*V* spectra of defects S_1_–S_4_ and the corresponding DFT simulations. (a–d) Comparison of the d*I*/d*V* spectra acquired right at the defect position and those from defect-free areas. (f,g,i,j,l,m,o,p) Spatially resolved d*I*/d*V* spectra of defects S_1_–S_4_, obtained along the dash arrows in (e,h,k,n), respectively. Tunneling parameters: (f,o) *V*_s_ = + 2.4 V, *I*_t_ = 300 pA, *V*_OSC_ = 24 mV; (i) *V*_s_ = + 2.4 V, *I*_t_ = 100 pA, *V*_OSC_ = 20 mV; (l) *V*_s_ = + 2.4 V, *I*_t_ = 100 pA, *V*_OSC_ = 24 mV; (g) *V*_s_ = – 1.5 V, *I*_t_ = 200 pA, *V*_OSC_ = 15 mV; (j) *V*_s_ = – 1.0 V, *I*_t_ = 100 pA, *V*_OSC_ = 10 mV; (m) *V*_s_ = – 1.4 V, *I*_t_ = 100 pA, *V*_OSC_ = 14 mV; (p) *V*_s_ = – 1.5 V, *I*_t_ = 100 pA, *V*_OSC_ = 15 mV. (q,r) DFT simulated topography images of S_1_ (q) and S_3_ (r) at a negative sample bias voltage. (s,t) DFT simulated LDOSs distribution across the defects S_1_ and S_3_, respectively. In-gap states that agree well with the experiments can be resolved.

Although the two types of antisite substitution are challenging to distinguish using STM topography images alone, the extra electronic states they introduce within the band gap are very different, making them easy to identify through d*I*/d*V* spectra. For the first type of atomistic configuration (S_1_ and S_2_, where S_1_ occurs in SAL and S_2_ in BAL), although their topography appearances are completely different, d*I*/d*V* spectra reveal almost identical in-gap states located right at the CBM energy of defect-free areas (Figure 5a,b,f,i), which introduce shallow n-type doping levels. These in-gap states are highly localized, expanding no further than the nearest unit cells. On the other hand, the second type of atomistic configurations (S_3_ and S_4_, with S_3_ in SAL and S_4_ in BAL), feature in-gap states approximately 0.6 eV lower than the CBM (Figure 5c,d,l,o), indicating deeper n-doping levels compared to those of S_1_ and S_2_. Furthermore, when measured under identical *V*_s_ and tunneling current *I*_t_, the d*I*/d*V* spectrum weight of the in-gap states of S_3_/S_4_ is much lower than that of S_1_/S_2_.

The main difference between the spectra of S_1_ and S_2_ is found in their behavior under negative sample bias voltages. At the VBM, the LDOS at the center of S_1_ is suppressed, while S_2_ displays almost no apparent features. This behavior can be attributed to the atomistic configuration of the antisite defect. Specifically, when the substitution happens at the SAL, the substituted Se atom is positioned significantly lower than the surrounding Sn atoms in this layer, resulting in a reduced LDOS at S_1_. In contrast, when the substitution happens in the BAL, the height of the atoms in the SAL is just slightly affected, which explains the indistinct feature in the LDOS at S_2_. Similarly, a comparable feature is observed in the spectra of S_3_ and S_4_.

It should be noted that the whole spectra of S_1_ is shifted downward by 0.1 eV compared to the other defects. This shift occurs because the spectra of S_1_ was acquired from a SnSe nanoplate grown on monolayer graphene, while those of S_2_∼S_4_ were derived from nanoplates on bilayer graphene. This energy shift indicates the differences in the work functions between monolayer and bilayer graphene surfaces, which has been consistently observed in our experiments.

Our DFT calculations also support the interpretation of the dual configurations of antisite substitution defects, indicating that both configurations are at local energy minima. Notably, the formation energy of S_1_/S_2_ is 0.32 eV lower than that of S_3_/S_4_ (Table 2), implying that the S_1_/S_2_ configurations are more stable. Furthermore, the spatially and energetically resolved LDOS distribution of S_1_ and S_3_ reproduces the in-gap states, with those of S_1_/S_2_ being closer to the CBM, aligning well with the experimental results.

Having characterized all 8 types of intrinsic point defects in monolayer SnSe, we calculated their formation energies and compared these theoretical results with the experimental data in Table 2. The formation energy of V_1_/V_2_ is 1 order of magnitude lower than that of V_3_/V_4_, which is consistent with the experimentally observed lower defect density of V_1_/V_2_. However, in practical samples, the density of V_2_ is 4 to 7 times higher than that of V_1_, despite being the same type of defect with identical formation energy in the calculations. This discrepancy is most likely from the influence of the grapitized SiC substrate, which modifies the chemical environment of the SnSe film from the bottom side and breaks the equivalence between V_1_ and V_2_. Using the Gundlach oscillation depicted in the d*z*/d*V* spectra,^39^ we can readily extract the difference in work functions between monolayer SnSe and the substrate (Figure S9). The comparatively higher work function of SnSe prompts electron transfer from graphene, leading to an accumulation of negative charge at the SnSe side of the interface. Theoretical studies have shown that the formation energy of Sn vacancies decreases as the VBM of SnSe moves farther from the Fermi energy,^23^ hence favoring their formation in BAL. Surprisingly, though the calculated formation energies of all the antisite substitution defects are negative, their densities are not significantly higher than those of the vacancy defects in experiments. (The antisite substitution defects were counted altogether in Table 2 because it was difficult to distinguish them merely from topography images.) This is probably due to our growth method of directly depositing SnSe molecules, during which most of the SnSe molecules do not break into atoms, limiting extra Se atoms. The negative formation energy of these defects can be explained by the phase diagram of Sn and Se elements. Given that a stable Se-richer phase, SnSe_2_, exists between these two elements, extra Se flux tends to form patches of SnSe_2_ inside SnSe, with antisite substitution defects serving as nucleation centers. In fact, people have observed the transition from SnSe films to SnSe_2_ during postannealing in a Se-rich environment, implying a lower formation energy of SnSe_2_.^40,41^

In addition, we have identified three types of extrinsic point defects: a Pb-substitution of a Sn atom in SAL (S_5_), originating from impurities in the evaporation material, and two types of surface adsorbates, derived from the residual gases in the vacuum chamber (A_1_ and A_2_).

**Figure 6:**
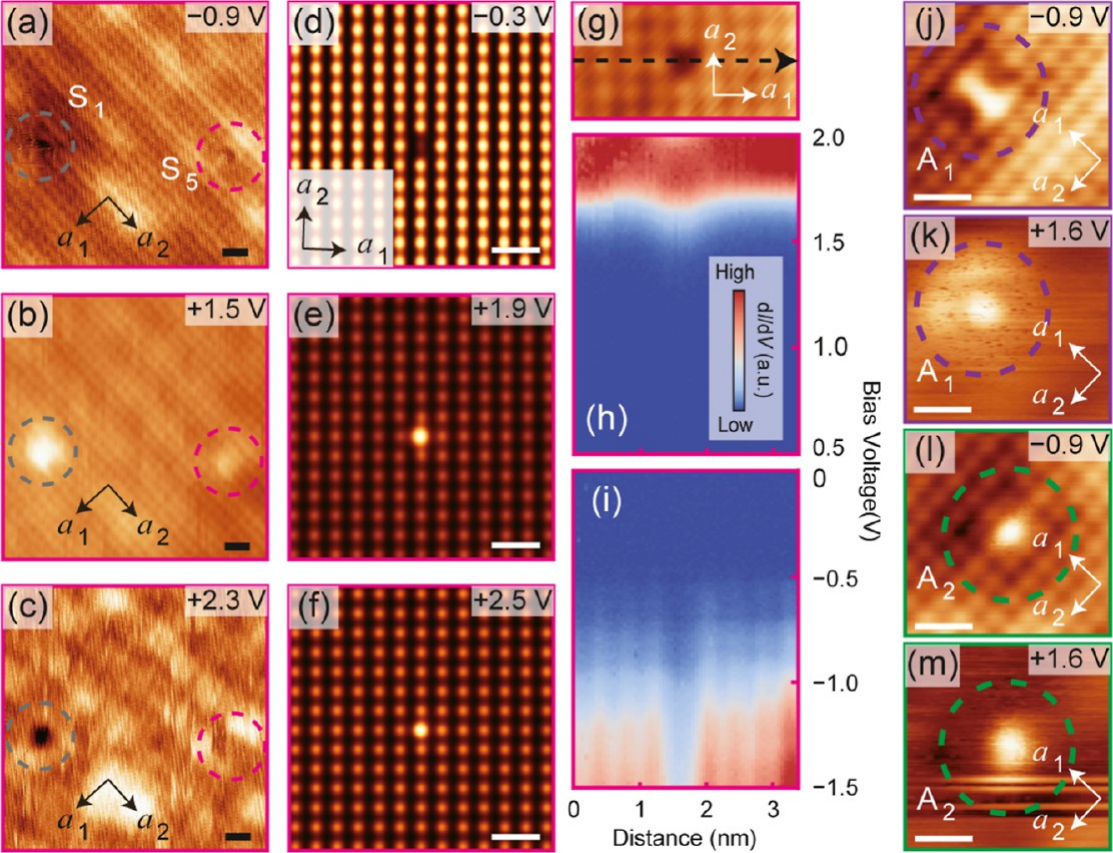
Three types of extrinsic defects. (a–c) Typical atomic-resolved STM topographic images of S_5_. The defects S_1_ and S_5_ are indicated by gray and magenta dash circles respectively. The tunneling parameters used are *V*_s_ = – 0.9 V, *I*_t_ = 10 pA; *V*_s_ = + 1.5 V, *I*_t_ = 10 pA; *V*_s_ = + 2.3 V, *I*_t_ = 10 pA, respectively. (d–f) Simulated atomic-resolved STM topographic images for S_5_. (g) Atomic-resolved STM topographic image of S_5_. (h,i) Spatially resolved d*I*/d*V* spectra obtained along the black dash arrow indicating in (g). Tunneling parameters: (h) *V*_s_ = + 2.4 V, *I*_t_ = 200 pA, *V*_OSC_ = 24 mV; (i) *V*_s_ = – 1.5 V, *I*_t_ = 200 pA, *V*_OSC_ = 15 mV. (j–m) STM images of typical adsorbates A_1_ and A_2_ which are indicated by purple and green dash circles respectively. The tunneling parameters used are *V*_s_ = – 0.9 V, *I*_t_ = 30 pA; *V*_s_ = + 1.6 V, *I*_t_ = 10 pA; *V*_s_ = – 0.9 V, *I*_t_ = 30 pA; *V*_s_ = + 1.6 V, *I*_t_ = 10 pA, respectively. All scale bars correspond to 1 nm.

The topographic features of S_5_ are similar to those of S_1_ and S_3_, except that the dip at negative *V*_s_ is shallower and the brightness at positive *V*_s_ is lower (Figure 6a–c), aligning with DFT calculations (Figure 6d–f). Considering that Pb-substitution for Sn in SnSe is isovalent, it naturally follows that S_5_ does not introduce extra in-gap states but merely modifies the structure of the band edges at the CBM and the VBM slightly (Figure 6g). Although the specific doping atom cannot be directly identified from the spectra, Pb doping is the most probable cause because (i) no impurities listed on the datasheet of the SnSe granules used for evaporation can lead to isovalent substitution; (ii) the MBE chamber was used for the growth of PbSe at the same time, potentially leading to slight cross-contamination. No sign of Pb-substitution defects were found in BAL, probably because it hardly affects the neighboring atoms and are thus not detectable.

Besides S_5_, two types of adsorbates positioned directly above a Sn atom were observed: one activating three atoms in a row (A_1_, Figure 6j) and the other activating only one atom (A_2_, Figure 6l) at negative *V*_s_. Interestingly, the brightness of the three atoms in A_1_ is not equal. The vector from the brightest to the dimmest atom is always parallel to the in-plane polarization of monolayer SnSe, making A_1_ a useful local indicator of polarization within SnSe. At positive *V*_s_, the appearances of A_1_ and A_2_ are similar. It is not yet known the exact types of molecules that are responsible for the adsorbates, but it can be assumed that A_1_ is from polar molecules like H_2_O or CO, while A_2_ is from nonpolar molecules like H_2_, N_2_, and O_2_. Further details about the extrinsic point defects can be found in the Supporting Information.

**Figure 7:**
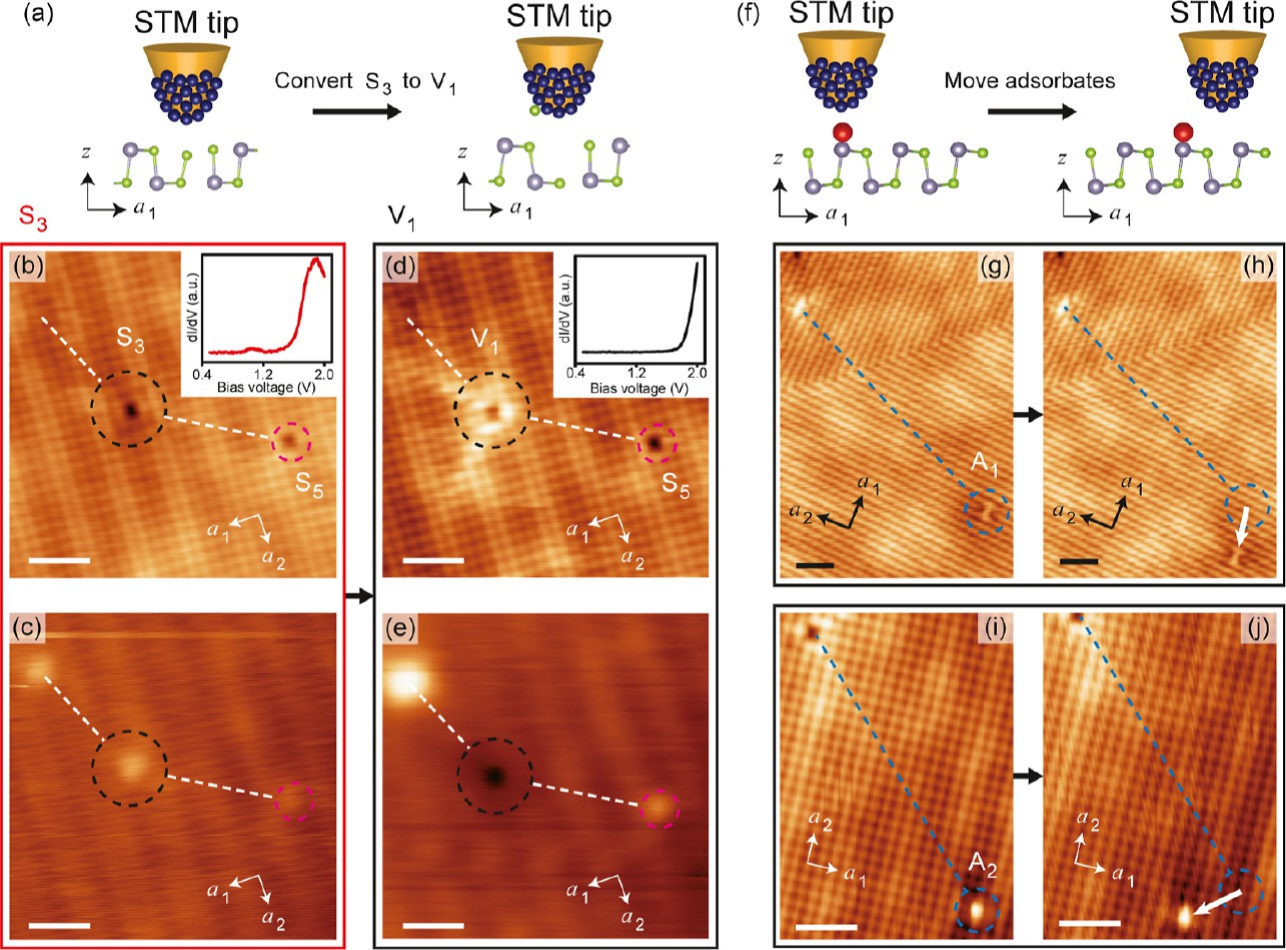
Manipulating point defects S_3_, A_1_ and A_2_ with a STM tip. (a) A schematic illustrating the process of converting S_3_ into V_1_. (b,c) Filled (b) and empty (c) state STM topography images before manipulating S_3_. The inset shows a typical d*I*/d*V* spectrum at S_3_. The S_5_ defect beside S_3_ serves as a local marker. The dash lines are guide for eyes to show the topography features around the S_3_. (d,e) Filled (d) and empty (e) state STM topography images after converting S_3_ into V_1_. The features marked by dash lines keep unchanged after the defect manipulation. (f) A schematic illustrating the process of relocating an adsorbate. (g,h) STM topography images before and after relocating an A_1_ defect. The dash line marks the distance from another V_2_ defect to the target A_1_ before moving, and the red solid arrow indicates the path of movement of A_1_. (i,j) STM topography images before and after relocating an A_2_ defect. Tunneling parameters: (b,d) *V*_s_ = – 0.9 V, *I*_t_ = 100 pA; (c,e) *V*_s_ = + 1.7 V, *I*_t_ = 10 pA; (g–j) *V*_s_ = – 0.9 V, *I*_t_ = 100 pA. All scale bars correspond to 2 nm.

We have not only identified the point defects in monolayer SnSe but also developed techniques to manipulate them. Figure 7a–e illustrate the process of converting a substitution defect S_3_ into a vacancy defect V_1_ through the interaction between the STM tip and the defect. Specifically, we position the tip above a defect S_3_ with tunneling parameters set at *V*_s_ = 1.6 V and *I*_t_ = 1.6 nA, then turn off the feedback loop and move the tip horizontally around the defect, and subsequently retract the tip from the surface. Following this operation, S_3_ is almost 100% converted into V_1_ by extracting the antisite Se atom out from the defect. Since the formation energy of S_3_ is lower than that of V_1_, this operation is a process of energy injection. Such manipulations can be applied to rationally adjust the local electronic states in monolayer SnSe. However, even when increasing *V*_s_ to over 4.0 V, we have not observed the conversion of S_1_ to any type of vacancy defect, implying that S_1_ maintains a stable configuration, while S_3_ is metastable, which consistent with their formation energies. Meanwhile, adsorbates A_1_ and A_2_ can also be relocated on the surface of monolayer SnSe following similar procedures (Figure 7f–j). Additionally, we have demonstrated the capability to remove a single Sn atom from a defect-free area of monolayer SnSe using the STM tip (Figure S11). Although the success rate of this operation currently stands at approximately 10%, it implies the potential to deliberately design patterns of vacancy defects through a series of manipulations with the STM tip.

## Conclusions

In conclusion, we have extensively investigated the point defects in monolayer SnSe grown by MBE, combining both STM studies and DFT calculations. Eight types of intrinsic defects were identified, including 4 types of vacancies and 4 types of antisite substitutions. The vacancy defects consist of the loss of either a single Sn atom or a vertically oriented SnSe molecule. Most of the vacancy defects exhibit noncentrosymmetric appearances that are consistent with the in-plane polarization in monolayer SnSe. Moreover, the density of atomic Sn vacancies in BAL is significantly higher than in SAL, likely due to the influence of the graphitized SiC substrate. Surprisingly, the antisite substitution defects, involving a Se atom replacing a Sn atom, exhibit in two distinct atomistic configurations. Despite their similar topographic appearances, the energies of the extra electronic states they introduce within the band gap of monolayer SnSe show significant differences. All substitution defects exhibit negative formation energies, yet their densities are limited by the growth method involving directly deposition SnSe molecules. Most interestingly, we have achieved nearly 100% success in converting an antisite substitution defect into a Sn vacancy using STM tip manipulation. Furthermore, we identified 3 types of extrinsic point defects, including a Pb-substitution of a Sn atom and 2 types of adsorbates. Our study has unambiguously revealed all observable point defects as well as their atomic and electronic structures, establishing methods for their manipulation, hence clarifying the influence of the point defects on the electronic structure of SnSe. The results of this study can be applied in the rational band engineering of both ultrathin and bulk SnSe for applications in thermoelectric, photovoltaic and nonvolatile logical devices.

## Materials and Methods

### Sample Growth

A monolayer of SnSe was grown on a graphitized 4H–SiC(0001) substrate using MBE under a base pressure of 1 × 10^–10^ mbar. The substrate preparation process, involving ultrahigh vacuum annealing, has been described in previous reports.^17^ SnSe molecules were evaporated from high-purity SnSe granules (99.999%, Alfa Aesar) contained in Knudsen cell, which was kept at 420 °C. The substrates were held at either room temperature or 100 °C during the deposition, and were subsequently annealed at 100 °C for 30 min to improve film quality.

### Low Temperature STM Characterization

The STM data were acquired with a Unisoku USM 1300 system directly linked to the MBE chamber. The as-prepared samples were characterized without exposure to the air. The measurements were performed at 4.2 K using mechanically sheared Pt/Ir alloy tips. Prior to measurements, both the topography and electronic states of the tip were calibrated on the surface of Ag(111) islands grown on a Si(111) substrate. The d*I*/d*V* spectra were obtained through lock-in technique, by applying a sinusoidal modulation at a frequency of 913 Hz.

### Manipulating the Defects

To convert a substitution defect S_3_ into a vacancy V_1_, the STM tip was first suspended above the target defect at *V*_s_ = 1.6 V and *I*_t_ = 1.6 nA. The feedback loop was then deactivated to fix the sample-tip distance. Subsequently, the tip was laterally moved (speed 1 nm/s) away from the defect along the ±*a*_1_ and ±*a*_2_ directions, before being retracted. To ensure a higher success rate, the tip movement were carried out repeatedly in all the four directions. Using this method, the probability of successfully converting S_3_ into V_1_ is nearly 100%. We have also attempted to manipulate S_1_ at a *V*_s_ up to 4.0 V, but no conversion was observed. At higher *V*_s_, the SnSe film could breakdown. The technique for relocating adsorbates was similar as above.

### DFT Calculations

We performed the calculations using the Vienna ab initio simulation package (VASP) code,^42,43^ with the projector augmented wave method^44^ employing the Perdew–Burke–Ernzerhof functional^45^ within the generalized gradient approximation to describe exchange correlation interactions. Defect structures were based on a 14 × 14 large supercell, with a vacuum space 12 Å to avoid interlayer interaction. All structures were relaxed until forces on each atom were smaller than 0.01 eV/Å, and the convergence criteria for electronic iteration was set to 10^–6^ eV. STM images were simulated based on partial charge densities from the VASP code, while LDOS for simulating d*I*/d*V* curves were calculated using the GPAW package.^46^ The formation energy is calculated by subtracting the total energy of the pristine bulk material from the total energy of the system containing the single defect.

## Supporting Information Available

Ferroelectricity in monolayer SnSe, buckled lattice in SnSe, V_2_ and S_2_ lattice site assignment, DFT simulations of the defects, work function difference between SnSe and graphene, extrinsic defects—S_5_, A_1_ and A_2_, and creating V_1_ defect from a defect-free area (PDF)
