# Supplementary material for: Identification and Manipulation of Atomic Defects in Monolayer SnSe
Source: ACS Nano. 2024 Sep 5;18(37):25478–88. doi: 10.1021/acsnano.4c04789 (PMC11411721; doi:10.1021/acsnano.4c04789)
Supplement: Supplementary file 1 — nn4c04789_si_001.pdf [file nn4c04789_si_001.pdf]

Supporting Information

Identification and manipulation of atomic defects in monolayer SnSe

Chengguang Yue[1], Zhenqiao Huang[2], Wen-Lin Wang[1], Zi'Ang Gao[1], Haicheng Lin[1,*], Junwei Liu[2,*], Kai Chang[1,*]

1. *Beijing Academy of Quantum Information Sciences, Beijing 100193, China*
2. *Department of Physics, Hong Kong University of Science and Technology, Clear Water Bay, Hong Kong, China*

*Correspondence to: linhc@baqis.ac.cn; liuj@ust.hk; changkai@baqis.ac.cn

This Supporting Information includes:

1. Ferroelectricity in monolayer SnSe

2. Buckled lattice in SnSe

3. V_2 and S_2 lattice site assignment

4. DFT simulations of the defects

5. Work function difference between SnSe and graphene

6. Extrinsic defects − S_5, A_1 and A_2

7. Creating V_1 defect from a defect-free area

1. Ferroelectricity in monolayer SnSe

The in-plane ferroelectricity in SnSe monolayer is characterized by the distribution of bound charges along the edges of the SnSe islands (Fig. S1a). The dipole field induces upward and downward band bending at the edges resulting in negative and positive bound charges, respectively. Consequently, the polarization direction can be inferred from the brightness and darkness contrast on the STM topography images at the island's edges, which corresponds to the local density of states at the band edges. Consistent with the earlier studies [1], the lattice period of the armchair direction of graphene is close to $\boldsymbol{a}_2$ of SnSe (4.26 Å), thereby SnSe tends to orient with $\boldsymbol{a}_2$ parallel to the armchair direction of graphene, resulting in stripe-like moiré patterns. By combining the information of band bending directions with the stripe patterns, we can determine the directions $\boldsymbol{a}_1$ and $\boldsymbol{a}_2$. Additionally, Fig. S1b and c illustrate a 180-degree ferroelectric domain wall, indicated by the white arrows.

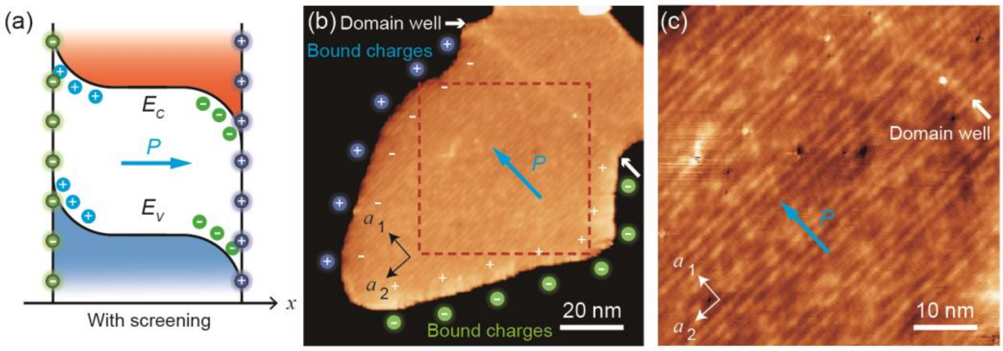

Figure S1. (a) Schematic of polarization induced band bending in SnSe. (b) STM topography of SnSe. The blue and green filled circles indicate the ferroelectric bound charges, while '+' and '−' represent the free charges. The bright and dark contrast at the edges results from band bending caused by the bound charges. (c) Atom-resolved image shows stripe patterns, indicating by the red dashed square in (b). The tunneling parameters were V_s = −0.9 V, I_t = 10 pA for (b) and V_s = −0.9 V, I_t = 50 pA for (c). The polarization direction is indicated by the blue arrows.

2. Buckled lattice in SnSe

To accurately pinpoint the locations of point defects, we initially verified the sublattice observed by STM across various sample bias voltages. Prior studies, utilizing atomic force microscopy, have demonstrated that the Sn sublattice at the surface of SnSe is higher than that of Se [2]. Notably, STM measurements consistently show that the Sn sublattice is the only observable sublattice [3]. To validate this observation, during the scanning process, we acquired atom resolved images at a designated sample bias voltage V_s for a predetermined period, and subsequently paused the scanning process, adjusted V_s, and resumed scanning until transitioned to another V_s. The applied V_s values are annotated on the right side of the images in Fig. S2. Consistent with prior research [3], we observed only Sn sublattice under different V_s.

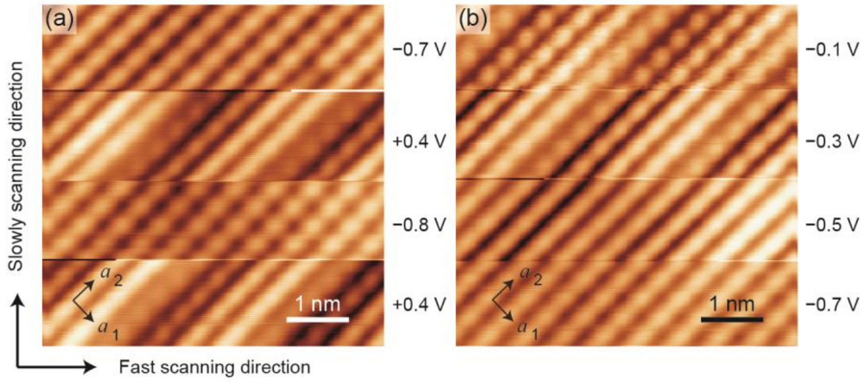

Figure S2. (a) A continuous STM topography image. The polarity of sample bias voltages was reversed during scanning. (b) Topography image of filled states at different sample bias voltages.

3. V_2 and S_2 lattice site assignment

To precisely determine the location of V_2, we conducted constant height mode dI/dV mapping at various sample bias voltages: $V_s = -0.8$ V, -0.9 V, -1.0 V, and -1.1 V, as presented in Fig. S3a-d, respectively. Notably, a distinct state emerges at the center of four nearest Sn atoms when the V_s is set to -1.0 V and -1.1 V. This observation suggests that V_2 sits at the Se site within the surface atomic layer (SAL) and contributes a defect state at these specific energy levels. These findings are consistent with the spatially resolved dI/dV spectra depicted in Fig. 3j.

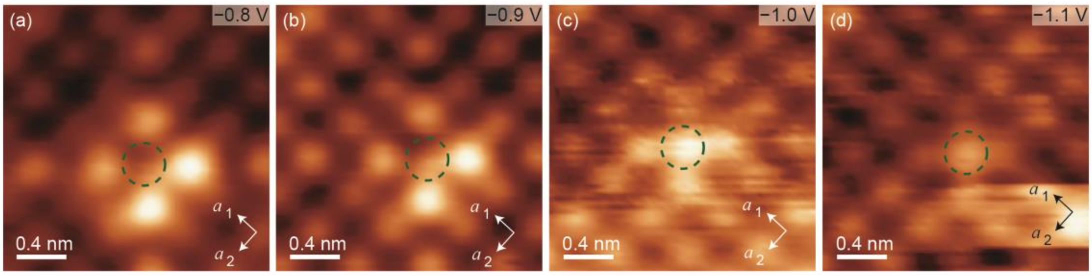

Figure S3. (a-d) dI/dV images of the defect V_2 acquired in constant height mode. The position of the intense occupied state is labeled by green dashed circles. The feedback loop was turned off at the tunneling current setpoint of 100 pA for all measurements. The sample bias voltages are indicated in the upper right corner of the panels.

To accurately determine the location of S_2, we took advantage of a nearby defect, S_5, which is an extrinsic defect that will be discussed later, as a reference. Fig. S4 clearly demonstrates the characteristic features of both S_2 and S_5. Specifically, in Fig. S4d, the precise location of S_5 is indicated. Additionally, Fig. S4b and S4c highlight the relative positions between S_2 and S_5. By combining the information from these figures, we were able to conclusively identify the location of S_2, which resides at the Sn position within the bottom atomic layer (BAL).

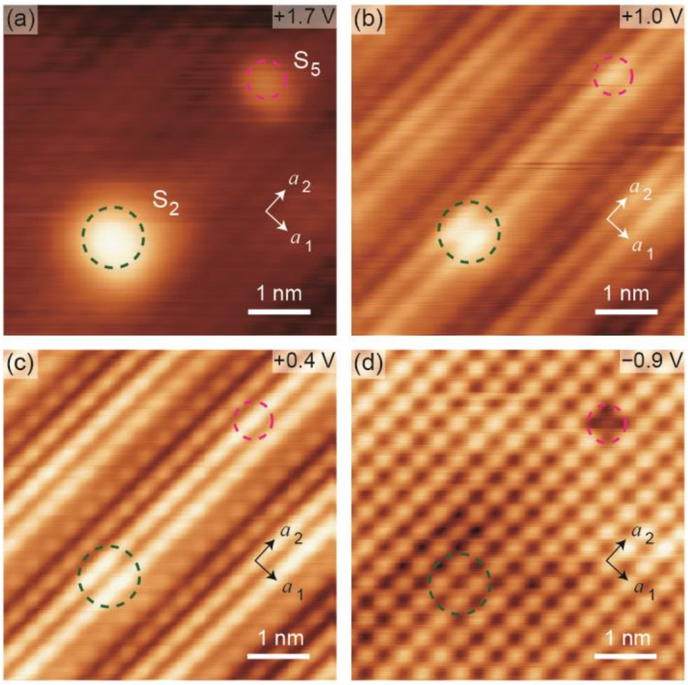

Figure S4. (a-d) Atom-resolved STM topography images of defect S_2 under different sample bias voltages. S_2 and S_5 are indicated by green and magenta dashed circles, respectively. The tunneling current is 50 pA for all and the sample bias voltages are indicated on the panels.

4. DFT simulations of the defects

The atom-resolved STM topography images were simulated by utilizing partial charge densities derived from the VASP code [4]. To establish a direct comparison with experimental findings, we selected specific sample bias voltages for generating these images. Additionally, the local density of state (LDOS) calculations for simulating dI/dV spectra were carried out using the GPAW package [4].

To compare with the experimental outcomes of vacancy defects V_1 to V_4, we simulated the STM topography images of both filled and empty states, along with their respective LDOS spectra (Fig. S5).

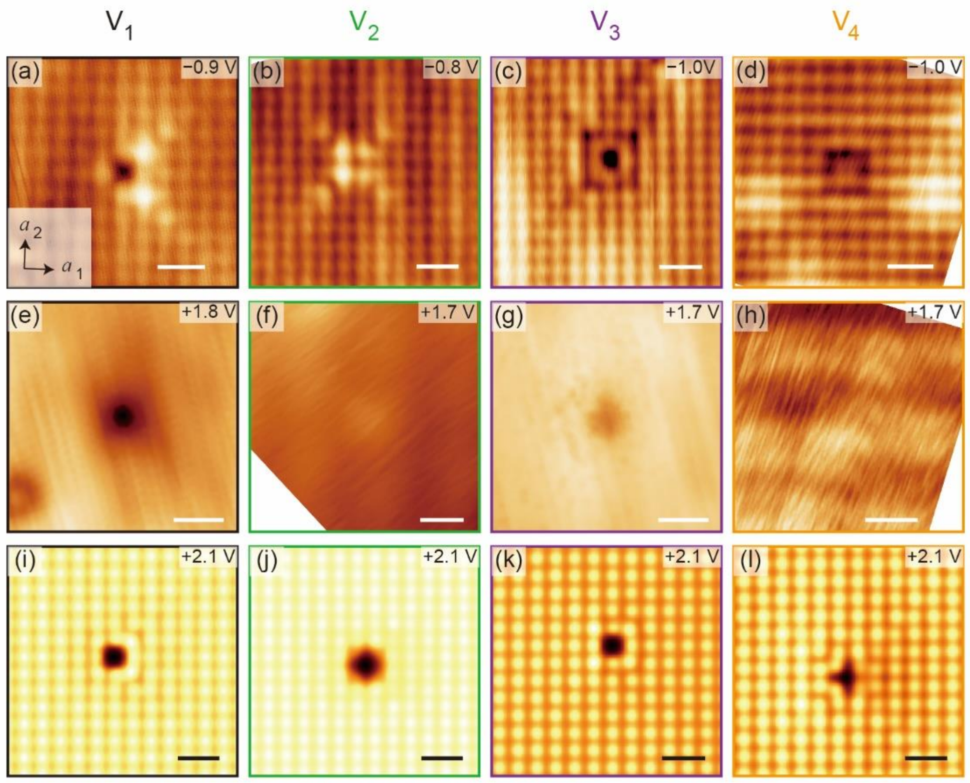

Figure S5. (a-h) Experimental and (i-l) simulated atom resolved STM topography images of all types of vacancies. The corresponding V_s is indicated on each panel. The I_t for each image is as follows: 10 pA for (a, c, d, f) and 30 pA for (b, e, g, h). All scale bars correspond to 1 nm.

Furthermore, Fig. 3f-p and Fig. S6 display the experimental and simulated spatially resolved d*I*/d*V* spectra for these defects.

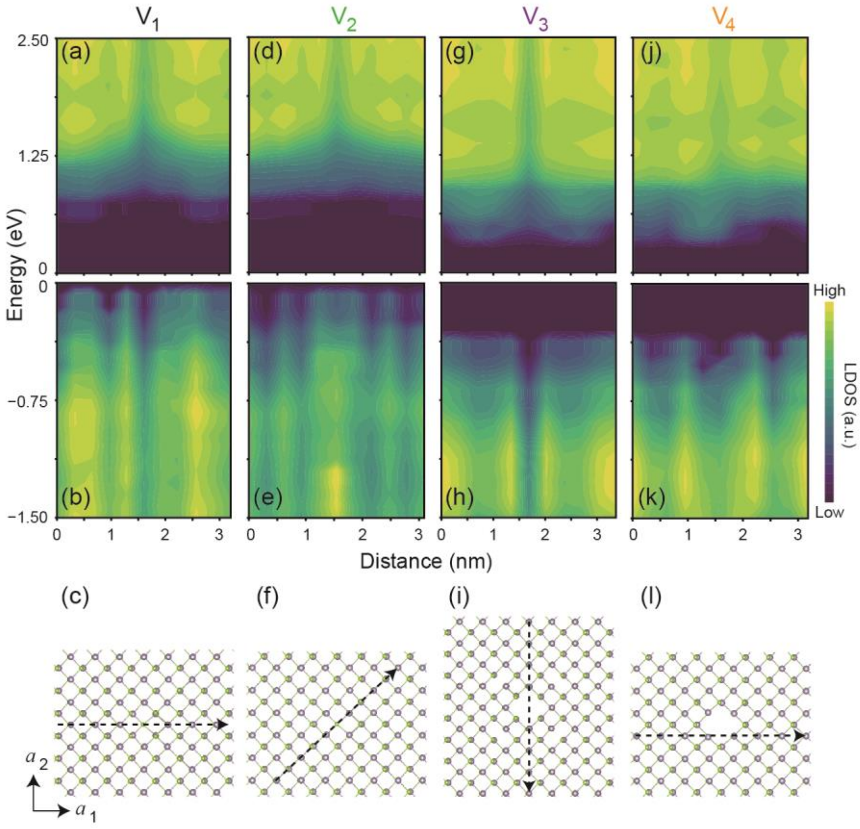

Figure S6. The simulated spatially resolved d*I*/d*V* spectra crossing V_1, V_2, V_3, and V_4, respectively. The spectra in (a, b), (d, e), (g, h), and (j, k) are acquired along the high-symmetry directions indicated by the dashed arrows in c, f, i, and l, respectively.

The experimental STM topography images of the intrinsic substitution defects S_1 to S_4 are presented in Fig. S7. Since S_1 and S_2, as well as S_3 and S_4, are the same type of antisite defects except that they appear in the SAL and BAL respectively, we simulated only the STM topography images of S_1 and S_3, as depicted in Fig. S8 and Fig. 4l-n. Additionally, the spatially resolved d*I*/d*V* spectra for these defects are shown in Fig. 5s and 5t.

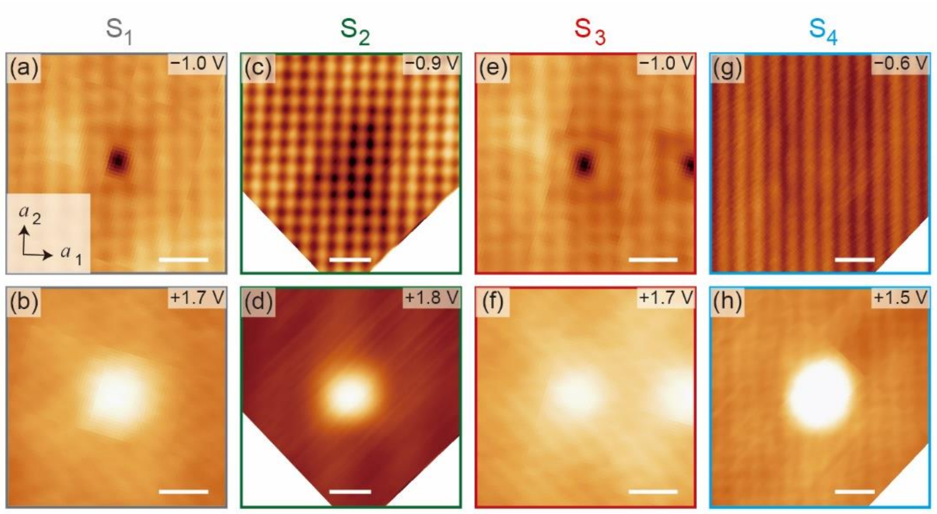

Figure S7. Atom resolved STM topography images of S_1 ~ S_4. The V_s values are indicated on the panels. The I_t for each image is as follows: 30 pA for (a, d, e), 10 pA for (b, f, g, h), and 50 pA for (c). All scale bars correspond to 1 nm.

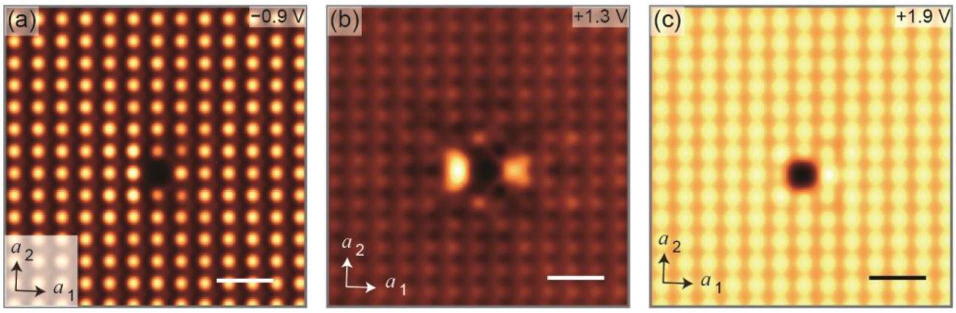

Figure S8. Simulated STM images of S_3. (a-c) The V_s values used in simulation are −0.9 V, +1.3 V, and +1.9 V, respectively. All scale bars correspond to 1 nm.

5. Work function difference between SnSe and graphene

Using Gundlach oscillations [5], we compared the work functions of SnSe and the graphene substrate. By numerically differentiating the z-V_s curve (acquired at a constant tunneling current), we obtained the (dz/dV)-V_s curve exhibiting the oscillations. Notably, the peak energies in the SnSe region are systematically higher than those in the graphene regions, with the exception of the zeroth-order peak (Fig. S9a). This consistent energy shift, observed between peaks of the same order in SnSe and the substrate, amounts to 0.58 eV in average, as depicted in Fig. 9b. Based on these observations, we conclude that the work function of SnSe is indeed higher than that of graphene substrate. The deviation of the zeroth-order is likely because the shape of the tunneling potential barrier is distorted from triangular at low bias voltages, which is common in Gundlach oscillation measurements [5].

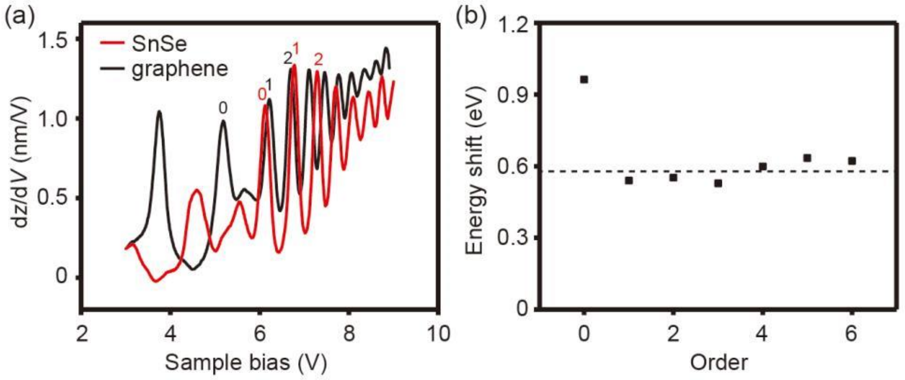

Figure S9. (a) The (dz/dV)-V_s curves of both the SnSe and graphene regions. The first few orders of Gundlach oscillations are highlighted on the peaks. (b) The energy shift between peaks of the same order in SnSe and the substrate as a function of the peak order shown in (a). The dashed line indicate the average peak energy shift except that of the zeroth-order peak.

6. Extrinsic defects − S_5, A_1 and A_2

To establish that S_5 is indeed a Pb-substituted Sn defect (Pb_{Sn}), we conducted a controlled experiment by co-depositing PbSe and SnSe to synthesize $Sn_{1-x}Pb_xSe$, as illustrated in Fig. S10a. The topography features of doped Pb atoms, whose density gradually increases as the flux of PbSe is tuned up, are identical as those of S_5 defects in the SnSe monolayers. This observation backs the hypothesis that S_5 is a Pb_{Sn} defect. To further elucidate the nature of the Pb_{Sn} defect, we undertook simulations of spatially resolved dI/dV spectra along the $\boldsymbol{a}_1$ direction, as presented in Fig. S10d.

The dI/dV spectra for the absorbates A_1 and A_2 are presented in Fig. S10e and f, respectively. For A_1, spatially resolved dI/dV measurements reveal the presence of in-gap states, specifically located around $V_s = +1.5$ V and $V_s = -0.5$ V. At A_2, we observe a notable enhancement in the LDOSs in the valence band, as depicted in Fig. S10f. When applying positive V_s, no significant alterations in the electronic state were observed.

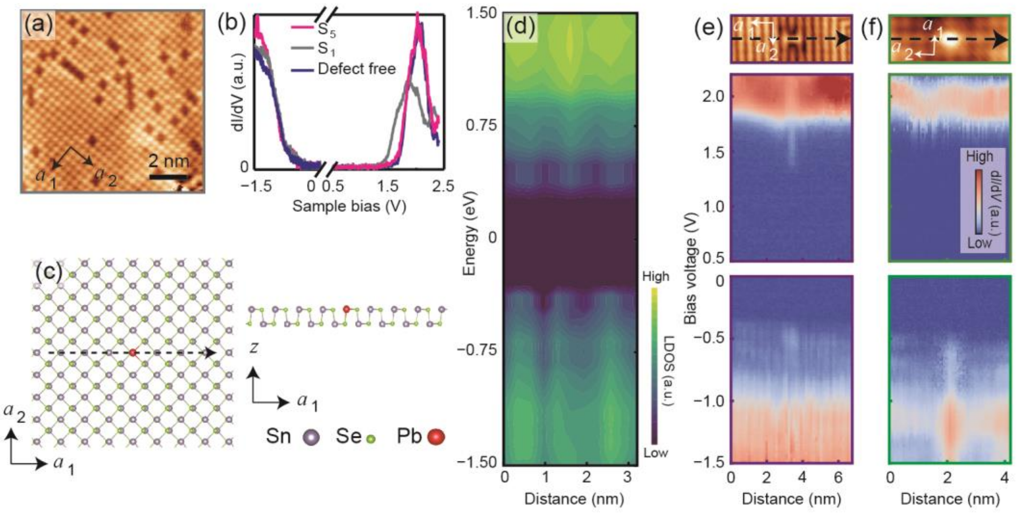

Figure S10. Three types of extrinsic defects. (a) Atom resolved topography image of $Sn_{1-x}Pb_xSe$. The tunneling parameters used are V_s = −1.6 V, I_t = 50 pA. The PbSe flux was maintained low to generate separated Pb substitution defects. (b) dI/dV spectra at S_5 and S_1, in comparison with that of the defect free area. (c) The atomic structure of defect S_5. (d)

Simulated spatially resolved LDOS of S_5. The acquisition path is indicated by the dashed arrow in (c). (e, f) Spatially resolved d*I*/d*V* spectra of A_1 and A_2, obtained along the dash arrows in the top panels. Spectra setpoints: (e) V_s = +2.2 V, I_t = 100 pA, V_{OSC} = 22 mV for positive V_s and V_s = −1.6 V, I_t = 200 pA, V_{OSC} = 16 mV for negative V_s; (f) V_s = +2.4 V, I_t = 100 pA, V_{OSC} = 24 mV for positive V_s and V_s = −1.5 V, I_t = 100 pA, V_{OSC} = 15 mV for negative V_s.

7. Creating V_1 defect from a defect-free area

By employing a more aggressive technique, we are able to remove an Sn atom from a defect-free area, resulting in the creation of a V_1 defect. First, the STM tip is suspend above the targeted atom at V_s = 1.6 V and I_t = 1.8 nA, where the feedback loop is turned off. Subsequently, the tip was moved vertically towards the film by 0.3 nm and then retracted. This operation has not been developed as mature as converting S_3 to V_1. Its success rate is merely at the level of 10%. Nevertheless, this route has been proved to be feasible. Figure S11 illustrates the corresponding STM images obtained both before and after the manipulation.

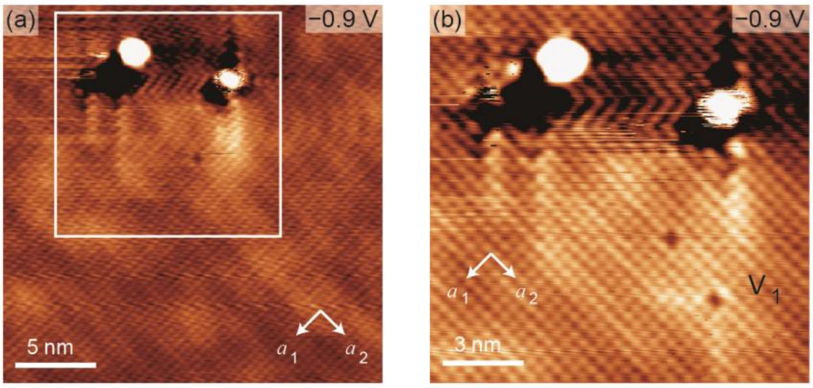

Figure S11. Creating a V_1 defect from a defect-free area in monolayer SnSe. The STM topography images were acquired before (a) and after (b) extracting one Sn atom from the SnSe lattice. The scanning area of (b) corresponds to the white box in (a). The tunneling parameters used were both V_S = −0.9 V, I_t = 100 pA.

References

1. Chang, K.; Küster, F.; Miller, B. J.; Ji, J.-R.; Zhang, J.-L.; Sessi, P.; Barraza-Lopez, S.; Parkin, S. S. P. Microscopic Manipulation of Ferroelectric Domains in SnSe Monolayers at Room Temperature. *Nano Lett.* **2020**, *20* (9), 6590-6597.

2. Morales, J.; Sánchez, L.; Santos, J. Structural Visualization of α-SnSe by Atomic Force Microscopy. *J. Solid State Chem.* **1999**, *148* (2), 513–516..

3. Kim, S.; Duong, A.-T.; Cho, S.; Rhim, S. H.; Kim, J. A Microscopic Study Investigating the Structure of SnSe Surfaces. *Surf. Sci.* **2016**, *651*, 5-9.

4. Enkovaara, J.; Rostgaard, C.; Mortensen, J. J.; Chen, J.; Dułak, M.; Ferrighi, L.; Gavnholt, J.; Glinsvad, C.; Haikola, V.; Hansen, H. A.; Kristoffersen, H. H.; Kuisma, M.; Larsen, A. H.; Lehtovaara, L.; Ljungberg, M.; Lopez-Acevedo, O.; Moses, P. G.; Ojanen, J.; Olsen, T.; Petzold, V.; Romero, N. A.; Stausholm-Møller, J.; Strange, M.; Tritsaris, G. A.; Vanin, M.; Walter, M.; Hammer, B.; Häkkinen, H.; Madsen, G. K. H.; Nieminen, R. M.; Nørskov, J. K.; Puska, M.; Rantala, T. T.; Schiøtz, J.; Thygesen, K. S.; Jacobsen, K. W. Electronic Structure Calculations with GPAW: A Real-Space Implementation of the Projector Augmented-Wave Method. *J. Phys. Condens. Matter* **2010**, *22* (25), 253202.

5. Lin, C. L.; Lu, S. M.; Su, W. B.; Shih, H. T.; Wu, B. F.; Yao, Y. D.; Chang, C. S.; Tsong, T. T. Manifestation of Work Function Difference in High Order Gundlach Oscillation. *Phys. Rev. Lett.* **2007**, *99* (21), 216103.
